# Uterine Cavity Irrigation With Office Hysteroscopy During Ovarian Stimulation for IVF: A Randomized Controlled Trial

**DOI:** 10.3389/fendo.2022.778988

**Published:** 2022-02-09

**Authors:** Marzieh Ghasemi, Ashraf Aleyasin, Human M. Fatemi, Faezeh Ghaemdoust, Mahnaz Shahrakipour

**Affiliations:** ^1^ Pregnancy Health Research Center, Department of Obstetrics and Gynecology, Zahedan University of Medical Sciences, Zahedan, Iran; ^2^ Department of Infertility, Faculty of Medicine, Shariati Hospital, Tehran University of Medical Sciences, Tehran, Iran; ^3^ In-Vitro-Fertilisation (IVF) Department, Assisted Reproductive Technology (ART) Fertility Clinics, Abu Dhabi, United Arab Emirates, Clinical Laboratory, Assisted Reproductive Technology (ART) Fertility Clinics, Abu Dhabi, United Arab Emirates; ^4^ School of Medicine, Tehran University of Medical Sciences, Tehran, Iran; ^5^ Department of Biostatistics, Zahedan University of Medical Sciences, Zahedan, Iran

**Keywords:** IVF cycle, uterine cavity irrigation, office hysteroscopy, endometrial receptivity, implantation

## Abstract

**Objective:**

This was a non-blinded randomized controlled study to evaluate whether endometrial irrigation *via* office hysteroscopy during the early follicular phase would lead to a higher level of live birth rates compared to no irrigation in the fresh embryo transfer cycle.

**Method:**

The study was conducted in Tehran university of medical sciences from June 2015 to June 2016. women under the age of 40 with primary infertility without history of previous IVF/ICSI or hysteroscopic examination, were included. Controlled ovarian hyperstimulation was done. Hysteroscopy was performed in the early mid-follicular phase of a stimulation cycle (day 5-7) with a vaginoscopy approach and saline irrigation in hysteroscopy group. Embryo-transfer was done in the same cycle.

**Results:**

228 patients completed their participation in the study. In the fresh cycle, clinical pregnancy rate was 46% in the hysteroscopy group and 40.43% in the control group. (*p-value= 0.326, RR= 1.16 [95%CI: 0.862 to 1.56]*). Live birth rate was 41.28% in the hysteroscopic group and 31.93% in the control group (*p-value=0.143, RR= 1.293 [95%CI: 0.916 to 1.825]*). For those patients having surplus cryopreserved embryos, after 2 months, a second embryo transfer was performed. The cumulative LBR was 44.05% in the hysteroscopic group and 32.25% in the control group (*p-value=0.029, RR= 1.368 [95%CI: 1.031 to 1.815], RD= 11.9% [95%CI: 1.2% to 22.3%] and NNT= 8 [95%CI: 4 to 85]*).

**Conclusion:**

The current study clearly demonstrated a significantly higher cumulative live birth rate in the intervention group.

**Clinical Trial Registration:**

[https://www.irct.ir/trial/19586], identifier IRCT2016011022795N2.

## Introduction

Notwithstanding the many recent advances in the field of ART, the chance of success is still very limited. Based on various publications, the implantation rate per embryo transfer is still around 35% (Planned transfers of cleavage-stage embryos: competency ≥ 25%; benchmark ≥ 35% - Blastocyst transfers: competency ≥ 35%; benchmark ≥ 60%) (1).

For a pregnancy to occur, a receptive endometrium, a functional embryo at blastocyst developmental stage and synchrony between the embryo and the endometrium is required (2). Failure to achieve receptivity and synchrony results in infertility and is a limiting factor for success in IVF treatment.

Among the papers and studies focusing on endometrial receptivity at the time of embryo transfer, Endometrial scratching in the cycle preceding IVF had been regarded as one of the most noteworthy methods that can affect the endometrial receptivity and probably improve the implantation rate (3). Although there are many explanations for the possible effect of endometrial injury on implantation, latest rigorous clinical trials and meta-analyses have claimed the inefficiency of this procedure on the implantation rate (4, 5). However, because of manifold methods and different timings and anatomical locations of the injury caused, it is still not completely evident whether any manipulation at any specific time of the cycle can improve the implantation results (6).

Alongside with endometrial scratching in the recent years, office hysteroscopy has been represented by many researchers as a means of improving uterine receptivity. Some successful outcomes have indeed been obtained through finding pathologies *via* hysteroscopy and treating them (7–9).

The current results also suggested that the benefit of hysteroscopy could extend beyond correction of uterine pathology. Some studies demonstrated that irrigation of the uterine cavity might even change the uterine environment and have a positive impact on uterine receptivity and the following implantations as well (10–12). Moreover, OH (Office Hysteroscopy), by dilatating the cervix would facilitating the subsequent embryo transfer and possibly by a total endometrial irrigation, create an aseptic endometrial inflammation (7).

Most of the aforementioned studies evaluated different aspects of using hysteroscopy or scratching and their effect on implantation. However, there are scarce data to date on the value of irrigation with hysteroscopy during the first IVF attempt. The aim of this study is to verify the role of uterine irrigation through hysteroscopy during the follicular phase, aiming to improve IVF outcomes in first IVF cycle candidates.

## Materials and Methods

The present study was conducted from June 2015 to June 2016 in the IVF Centre of Shariati hospital, Tehran University of Medical Sciences. It is a non-blinded randomized controlled trial which aimed to assess the possible benefit of hysteroscopic uterine irrigation, prior first IVF cycle, on the reproductive outcomes.

The study protocol was approved by the IRCT and ethics committee of Tehran University of Medical Sciences (Ethics committee reference number: IR.TUMS.REC.1394.1598). Trial registration number: IRCT2016011022795N2

### Participants

248 patients with primary infertility were recruited for this study. Participants with regular menstrual cycle (regular menses defined as a duration of 24-35 days), age ≤ 40 years, BMI between 19- 30, without any prior hysteroscopic examination or previous IVF or ICSI, normal Transvaginal sonography (TVS) in the last month and hysterosalpingography (HSG) (between 6-24 months), who were scheduled for the first IVF cycle, were included.

**Figure 1:**
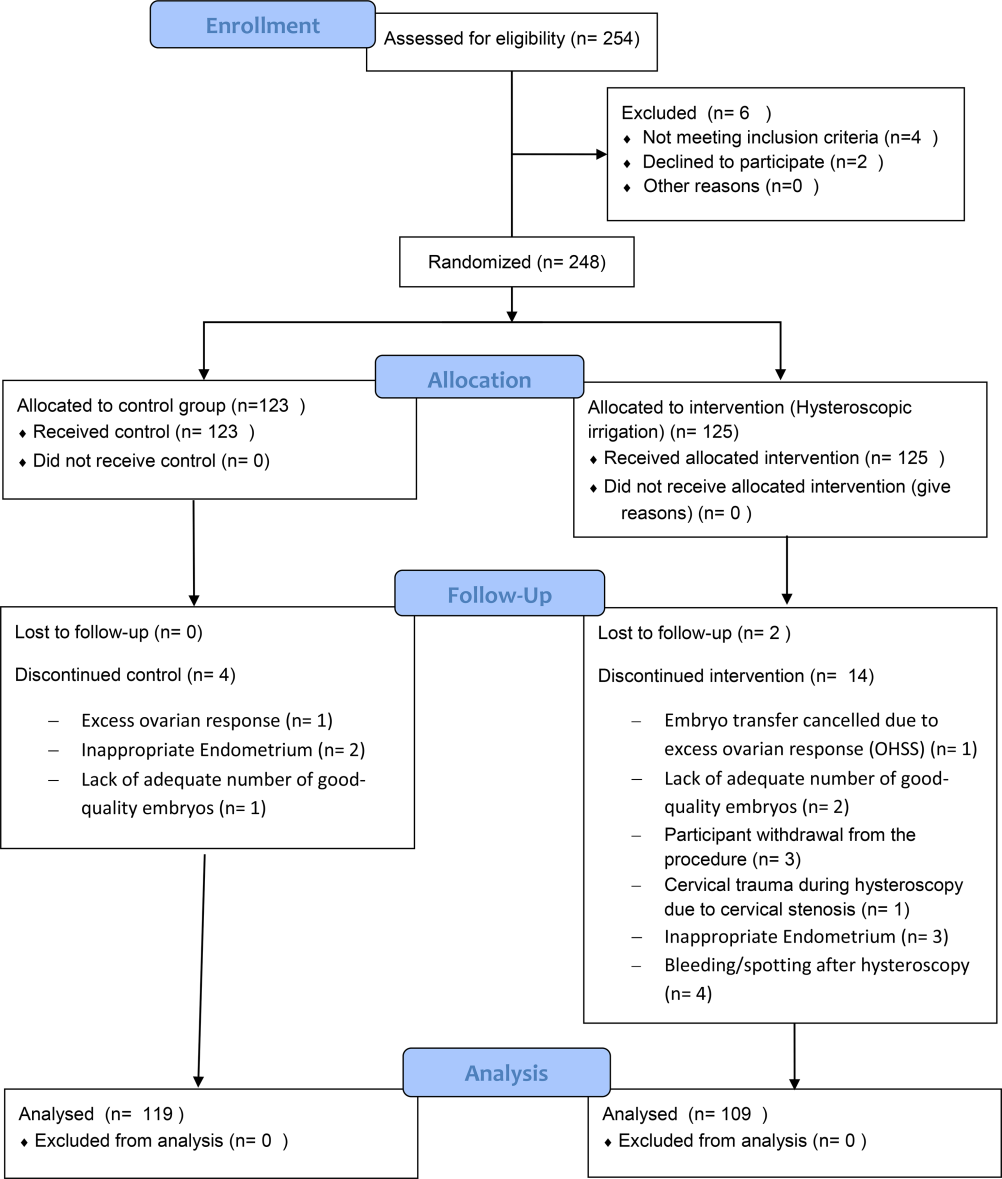
CONSORT flow diagram of the study.

Exclusion criteria were determined as recurrent miscarriage (3 or more miscarriages), intermenstrual bleeding, any doubt about uterine cavity abnormality, azoospermia, patients with poly cystic ovary syndrome (PCOS) or endometriosis AFS 3/4, hydrosalpinx, ovarian cysts and cancellation of the same cycle for any reason. Moreover, participants were excluded, if there were any difficulties with the hysteroscopy procedure, such as bleeding post procedure or detecting uterine cavity abnormalities or inability to perform the procedure (and necessity to use Hegar dilator in order to dilatated the cervix (**Figure 1**).

Prior entering the study, the purpose and process of the study was clearly explained to all participants and they all gave written informed consent. The patients had the right to quit the study at any time for any reason. Physicians and other healthcare workers involved in this study were all respectful to the principles of Good Clinical Practice and the Declaration of Helsinki.

All patients participating in the study underwent the standard infertility work-ups, consisting of the medical history, physical examination, hormonal status and their spouses’ semen analysis.

The patients who accepted to join the study and met all the relevant criteria, were randomized into two groups by a web-based randomization program which was based on block randomization: group I- with hysteroscopic irrigation in the IVF cycle (intervention) and group II-without hysteroscopy (control).

### Procedures

#### Ovarian Hyper Stimulation and Embryo Transfer Methods

Controlled ovarian hyper stimulation was done in an antagonist regimen in the two groups with recombinant FSH (Gonal-f, Merck-Sereno) starting on day 2 of the cycle at a dose of 150 IU daily for the first 6 days, the dosage was adjusted according to the patient’s individual ovarian response. The GnRH antagonist (cetrorelix, Actoverco) was initiated when the leading follicles had a diameter 14 mm. The final oocyte maturation was achieved by the administration of HCG (choragun) I.M. 10000 IU as soon as 3 follicles reached 17mm in diameter. Oocyte retrieval was carried out 36 hours post HCG administrations. Embryo transfer was performed on day 3 after oocyte retrieval and only two grade 1 embryos (cleavage) were transferred. Luteal phase supplementation consisted of suppository progesterone (supp Cyclogest, Actoverco 400 mg BID) after oocyte retrieval and was continued till the 8th week of gestation.

#### Hysteroscopy

Hysteroscopy and irrigation of uterine cavity with a large amount of saline solution (200-300 cc) was perform for the intervention group in the early mid-follicular phase of a stimulation cycle (day 5-7). It was implemented with a five French working channel and a 30° direction of view hysteroscope with a Vaginoscopic approach. The endocervical canal, uterine cavity, tubal orifices and endometrium were all inspected and recorded on standardized forms. In order to reduce inter-operator bias, all hysteroscopies were conducted by the same surgeon. Finally, participants in both groups underwent embryo transfer in the same cycle as the hysteroscopic irrigation (for intervention group).

#### Embryo Transfer

Morphology of the embryos were categorized as grade 1 based on Giorgetti-4-point-score (13). Embryo transfer for all participants were implemented after 3 days of fertilization based on standard protocols (14).

After 14 days of embryo transfer, beta-HCG was tested and if positive results were reported, ultrasonography was performed in order to confirm the clinical pregnancy. In case of pregnancy failure and existence of frozen embryos, they would be included for second transfer cycle.

### Inclusion Criteria to the Second Freezing Cycle

Workups for the second cycle started for participants who did not get pregnant after the embryo transfer: OCP was prescribed in the next menstrual cycle. After 5 days of OCP free interval the cycle, estradiol valerate 6mg/day (Aburaihanco 2 mg Tab) was administered for 10 days. At this point, if the endometrial thickness (ET)was not proper for the procedure (ET less than 8 mm), dose-adjustment was done (8 mg) and another ultrasonography was performed 3-5 days later to evaluate whether the ET is ready (If the endometrium did not reach that level, the participants would be excluded from the study). Once the endometrial thickness reached 8mm or more, progesterone was prescribed (supp Cyclogest, Actoverco 400 mg BID) and embryo transfer would start after completing the 3 days of progesterone administration (in the 4^th^ day). Embryo transfer was done by using COOK catheter. After 14 days of embryo transfer, beta-HCG was tested and if positive results were reported, ultrasonography was performed in order to confirm the clinical pregnancy in 6 weeks.

### Outcome Measures

The primary objective of the current study was live birth rate (LBR). Secondary outcomes were clinical pregnancy rate, Chemical pregnancy rate, Abortion rate and implantation rate (per cycle and per transfer). Pregnancy test was done 2 weeks after embryo transfer and positive tests were confirmed after a week. The aforementioned outcomes are calculated as indicated in the following section.

#### Definitions

Chemical (biochemical) pregnancy rate: beta-HCG serum level more than 25 mIU/ml in 2 weeks after embryo transfer
Clinical pregnancy rate: observing one or more gestational sacs around the age of 6 weeks by Ultrasound imaging
Live birth rate: any parturition after 27 weeks
Abortion rate: any pregnancy failure before the 20 weeks.
Implantation rate: number of gestational sacs observed divided in the number of embryos transferred

### Statistical Analysis

Sample size was calculated based on the observed IVF cycle outcomes and differences in outcomes from existing literature. Based on a study by Rama Raju et al. (15), clinical pregnancy rate considered 25% in control group and 40% in OH group. For the difference of 15% increase in CPR and a power of 80% and a one sided alpha error of 5%, 240 participants in total were calculated to be included. Considering the lost to follow-up in some patients, 254 women enrolled in the study statistical analysis was performed using the SPSS version 22 and web based EBM calculator.

Primary transfer results and secondary (freezing) transfer results were calculated separately. Thereafter, all transfers underwent a cumulative analysis which were evaluated both per transfer and per cycle.

In order to make sure that the randomization was properly implemented, patients and their ovulation induction characteristics, T-test or its non-parametric equivalent were done.

For comparing the pregnancy outcomes between the two groups chi-square test was applied. Relative Risk with 95% CI were also calculated. For statistical significance, P-value<0.05 was considered.

## Results

From 254 patients whose eligibility were assessed, 248 enrolled in the study and were randomly assigned to any of the intervention or control group (125 participants in Hysteroscopy group and 123 participants in control group). From the 125 participants in the hysteroscopy group, 16 were excluded due to different reasons and finally 109 participants from this group underwent embryo transfer. From 123 participants in control group, 4 were excluded and 119 participants underwent embryo transfer (**Figure 1**).

### Participant’s Characteristics

Relevant characteristics of participants were compared between these two groups. There were no significant differences in the Age, Weight, BMI and ovarian reserve (tested by FSH, AMH,AFC) between the two groups (**Table 1**).

**Table 1:** Patients’ Characteristics.

Variable	Intervention group (mean±SD)	Control group (mean±SD)	p-value
Age	29.90 ± 3.805	30.54 ± 4.20	0.233
Weight	63.84 ± 9.09	61.99 ± 8.97	0.115
FSH	4.52 ± 2.17	5.03 ± 2.32	0.094
LH	7.73 ± 4.78	7.57 ± 4.84	0.797
AMH	2.97 ± 1.22	3.37 ± 2.84	0.154
AFC	10.81 ± 2.67	10.68 ± 2.58	o. 709
TSH	2.03 ± 0.91	1.97 ± 1.01	0.637
BMI	24.19 ± 2.84	23.94 ± 3.52	0.545

### Cycle’s Characteristics and First Transfer Features

Characteristics of ovarian stimulation cycles were also compared between these 2 groups. Variables including duration of ovarian stimulation, gonadotropin dosage, number of follicles with 14 mm or more in diameter in the day of final oocyte maturation, number of retrieved oocytes, number of metaphase II oocytes and also number of yielded embryos were all compared. Number of GnRH antagonist administration. There were no significant difference between the 2 groups compared which indicates the similarity of baseline characteristics in both groups. Moreover, the number of transferred embryos, number of frozen embryos and the endometrial thickness did not reveal any significant difference between the two groups compared (**Tables 2A, B**).

**Table 2:** A) Cycles’ characteristics B) first transfer characteristics.

Variable	Intervention group (mean±SD)	Control group (mean±SD)	p-value
**A) Cycle's Characteristics**			
Duration of ovulation induction (day)	10.15 ± 0.812	10.25 ± 1.04	0.392
Gonadotropin dosage	2740.43 ± 638.25	2877.75 ± 724.50	0.052
Number of folicles ≥ 14 mm	12.58 ± 4.57	12.16 ± 3.68	0.428
Number of oocytes after pick up	12.88 ± 4.72	12.26 ± 5.12	0.327
Number of oocytes in Metaphase Il	8.82 ± 4.36	8.56 ± 4.67	0.657
**B) First transfer characteristics**			
Total Embryo	7.73 ± 4.07	7.29 ± 4.16	0.407
Number of embryo transferred	2.33 ± 0.90	2.44 ± 0.79	0.292
Number of embryo freezed	4.89 ± 3.84	4.54 ± 4.01	0.489
Endometrial thickness	8.18 ± 0.71	8.23 ± 0.71	0.622

### Fresh Embryo Transfer Results

In the fresh embryo transfer of the hysteroscopy group, 53% of the included patients had a positive beta-hCG as compared to 45.5% in the control group (*p-value=0.23, RR= 1.173 [95%CI: 0.9 to 1.527]*).

Clinical pregnancy rates have been 46% and 40.33% in the hysteroscopy and control group, respectively (p-value=0.32, *RR=* 1.16 [*95%CI*: 0.862 to 1.56]).

Live birth rates have been 41.28% in hysteroscopy and 31.93% in control group, respectively (*p-value=0.143, RR= 1.293 [95%CI: 0.916 to 1.825]*)

From 58 patients who got pregnant in the hysteroscopic irrigation group, 45 resulted in live births (41%) with an abortion rate of 22.41%. In the control group, out of 54 women who got pregnant, 38 of them had live births (31%), with an abortion rate of 25.92%. (p-value: 0.664)

The implantation rates were 22.85% and 18.62% in hysteroscopy ad control groups, respectively (p-value: 0.206).

While none of the outcome measures related to fresh embryo transfer were statistically significant, 9.4% difference was showed in live birth rates between the two groups compared, in favour of the hysteroscopy group (**Table 3**)

**Table 3:** Fresh Embryo Transfer results.

Variable	Intervention group (109)	Control group (119)	p-value	RR [95%Cl]
Chemical Pregnancy Rate	58 (53%)	54 (45.4%)	0.237	1.173[ 0.9 to 1.527]
Clinical Pregnancy Rate	51 (46%)	48 (40.33%)	0.326	1.16 [0.862 to 1.56]
Live Birth Rate	45 (41.28%)	38 (31.93%)	0.143	1.293 [0.916 to 1.825

live birth risk difference: 9.4% [95%Cl: -3.1% to 21.5%].

### Second Transfer Characteristics

Among participants, who did not get pregnant in the first transfer and had frozen embryos available, underwent a subsequent cryo-embryo transfer.

Characteristics for this second transfer are presented in **Table 4**. Variables like number of embryos transferred, endometrial thickness, day of embryo transfer were compared and did not reveal any significant differences.

**Table 4:** Second (Frozen) Transfer Characteristics.

Variable	Intervention group (mean±SD)	Control group (mean±SD)	p-value
Number of (frozen) embryos transferred	2.52 ± 0.50	2.67 ± 0.47	0.176
Endometrial thickness	8.23 ± 0.424	8.28 ± 0.84	0.755

### Frozen Embryo Transfer Results

34 participants from hysteroscopy group and 58 participants from the control group underwent a second embryo transfer procedure using frozen embryos. From the 34 patients in the hysteroscopy group, 21 had positive pregnancy test (61.76%) and in the control group, from the 58 participants, 25 (43.10%) had positive beta-hCG (*p-value=0.084, RR= 1.433 [95%CI: 0.964 to 2.131]*).

Clinical pregnancy rate was 52.94% and 36.20% in the hysteroscopy and control group, respectively (*p-value=0.12, RR= 1.462 [95%CI: 0.918 to 2.33]*).

Live birth rate had an average of 52.94% in the hysteroscopy group and 32.75% in the control group (p-value=0.06, *RR= 1.616 [95%CI: 0.994 to 2.628]*).

Abortion rate was 14.28% and 24% in the intervention and control group, respectively (p-value=0.40).

Implantation rate in hysteroscopy group was 18.62% compared to 16.12% in the control group (p-value= 0.117).

Similar to the first transfer, none of the outcome measures in the second transfer were significantly different between 2 groups. However, the live birth rate was 20% higher in the intervention group. Detailed results of this second transfer are presented in **Table 5**.

**Table 5:** Frozen Embryo Transfer Results.

Variable	Intervention group (34)	Control group (58)	p-value	RR [95%Cl]
Chemical Pregnancy Rate	21 (61.76%)	25 (43.10%)	0.084	1.433 [0.964 to 2.131]
Clinical Pregnancy Rate	18 (52.94%)	21 (36.20%)	0.117	1.462 [0.918 to 2.33]
Live Birth Rate	18 (52.94%)	19 (32.75%)	0.06	1.616 [0.994 to 2.628]

live birth risk difference: 20.2% [95%Cl: -0.5% to 39.1%].

### Cumulative Results

Cumulatively (**Table 6**), 143 and 177 transfers were conducted for the hysteroscopy and control group, respectively. Cumulative positive pregnancy rate was 55.24% in the hysteroscopy group and 44.63% in the control group (*p-value=0.050, RR= 1.238 [95%CI: 0.993 to 1.543]*) Clinical pregnancy in hysteroscopy group was 48.25% and in control group 38.98% (*p-value= 0.096, RR= 1.238 [95%CI: 0.963 to 1.59]*). Live birth rates were 44.05% and 32.20% in the hysteroscopy and control group, respectively (*p-value=0.029, RR= 1.368 [95%CI: 1.031 to 1.815]*). The difference in live birth rate between two groups was statistically significant in favour of the hysteroscopy group (*live birth risk difference: 11.9% [95%CI: 1.2% to 22.3%]*).

**Table 6:** Cumulative Pregnancy Outcomes.

A) per transfer	Variable	Intervention group (143)	Control group (177)	p-value	RR (95%Cl)
	Chemical Pregnancy Rate	79 (55.24%)	79 (44.63%)	0.05	1.238 [0.993 to 1.543]
	Clinical Pregnancy Rate	69 (48.25%)	69 (38.98%)	0.096	1.238 [0.963 to 1.59]
	Live Birth Rate	63 (44.05%)	57 (32.25%)	0.029	1.368 [1.031 to 1.815]
**B) per cycle (patient)**	**Variable**	**Intervention group (109)**	**Control group (119)**	**p-value**	**RR (95%Cl)**
	Chemical Pregnancy Rate	79 (72.47%)	79 (66.38%)	0.394	1.092 [0.919 to 1.297]
	Clinical Pregnancy Rate	69 (63.3%)	69 (57.98%)	0.493	1.092 [0.886 to 1.346]
	Live Birth Rate	63 (57.79%)	57 (47.9%)	0.173	1.207 [0.943 to 1.544]

Abortion rates were 20.25% in the intervention group and 25.31% in the control group (p-value=0.448). Implantation rate in the hysteroscopy group was 23.22% as compared to 17.78% in the control group (p-value= 0.05).

Cumulatively (**Table 6**), chemical pregnancy rate was 72.47% among the hysteroscopy group and 66.38% in the control group (*p-value=0.394, RR= 1.092 [95%CI: 0.919 to 1.297]*). Clinical pregnancy in hysteroscopy group was 63.3% and in control group was about 57.98% (*p-value= 0.493, RR= 1.092 [95%CI: 0.886 to 1.346]*). Live birth rates were 57.79% and 47.9% in the hysteroscopy and control group respectively (*p-value=0.173, RR= 1.207 [95%CI: 0.943 to 1.544]*). Neither of the per cycle results were statistically significant. However, live birth rate was 9.9% higher in the intervention group (*live birth risk difference: 9.9% [95%CI: -3% to 22.4%]*).

## Discussion

In this study, hysteroscopic irrigation did not show a significant improvement of the Live birth rate in the fresh cycle (*RR = 1.293 [95%CI: 0.916 to 1.825]*). However, taking the frozen cycle into account, this intervention clearly demonstrated a significantly higher cumulative live birth rate (*RR = 1.368 [95%CI: 1.031 to 1.815]*. despite being significant, since the sample size was not calculated regarding the cumulative results, we can not claim this significance to be important. However, these results might help in hypothesizing and conducting more rigorous similar studies with greater sample sizes and these future study and meta-analyses might show a noteworthy association.Moreover, the current study demonstrated that an atraumatic hysteroscopy during ovarian stimulation for IVF, doesn’t not harm the patient and significantly increases the cumulative livebirth rate

In the past, Endometrial Scratching (ES) was one of the most popular methods proposed to possibly improve the endometrial receptivity. However, recent meta-analysis and high-powered studies failed to demonstrate any benefit of ES on pregnancy outcome. (4, 5, 16, 17). The most notable explanation is that ES could be highly traumatic and might damage some areas of endometrium. However, this is not the case with hysteroscopy and irrigation. In the current study, no harm/trauma was caused to the endometrium. The uterine cavity was accessed just by the saline without any scratching or biopsy. We did so since it was presumed that taking a piece of endometrium might cause damage to the endometrium (4, 18) and might later lead to a disturbance for the implantation in the next transfer cycle. Karimzadeh et al. reported that performing ES in the same transfer cycle at the day of oocyte retrieval would have harmful effects on the outcomes (18).

Aside from being harmless, there are other hypothesis supporting the possible benefits of cavity irrigation: First, irrigation can mechanically remove detrimental anti-adhesive glycoproteins from the surface of the endometrium and subsequently improve the endometrial receptivity (11). Considering that hysterosalpingography can sometimes improve the pregnancy outcomes, a growing body of evidence supports the hypothesis that uterine flushing can improve fertilization by removing debris from tubes and also changing the production of cytokines (19).

Another explanation emphasizes the act of hysteroscopy itself as a diagnostic procedure; crossing the cervical canal with the tip of hysteroscope might lead to the lysis of cervical adhesions and might help in gathering information about the morphology of the cervical canal. These factors can facilitate the upcoming embryo transfer procedure (3, 20, 21).

Another plausible explanation suggested on all kinds of mechanical manipulations is that those acts can initiate changes in the immune system and gene expression in such a direction that could help to improve receptivity and implantation (20, 21). Inagaki et al. showed that in patients with recurrent implantation failure the level of MMP activity and cytokine concentrations had a different pattern in the lavage (22); the effect of irrigation on immunity-related factors and cytokines can probably help in reversing this pattern.

Moreover, it was also suggested that mechanical manipulation in the preceding cycle can be more effective since these changes require time in order to show the results and also intervention in the same cycle as embryo transfer can disturb the endometrium (21).

Theoretically, fluid infusion and irrigation of the uterine cavity could also be considered as a form of an “atraumatic global endometrial injury”. Some studies suggested the potential effect of immunomodulation triggered by uterine bathing using Lipiodol^®^ (23, 24). In contrast, some other trials found no difference in using pharmacologically neutral gels for uterine bathing (25, 26)

Salehpour et al. showed in their trial that intrauterine saline infusion using IUI catheter during IVF cycles could have a negative impact on pregnancy outcomes in RIF patients (27).

Throughout our study, this hysteroscopic irrigation method was well-tolerated and accepted by the patients in the intervention group. No case of endometritis was found.

Lack of blinding and small sample size were among the limitations of our study, given that an extra procedure like hysteroscopy might encourage the intervention group’s participants to cooperate more widely than the other group’s.

In conclusion, the current study could not show a significant difference in any of the pregnancy outcomes between the groups. Although there was a significantly higher cumulative per transfer live birth rate in the intervention group. Future, large RCTs are required to confirm the current findings.

## Data Availability Statement

The raw data supporting the conclusions of this article will be made available by the authors, without undue reservation.

## Ethics Statement

The studies involving human participants were reviewed and approved by The Ethics committee of Tehran University of Medical Sciences- IR.TUMS.REC.1394.1598. The patients/participants provided their written informed consent to participate in this study.

## Author Contributions

AA: designing the study, patient selection, supervising the study, revising the manuscript. HF: helping in designing the study and revising the manuscript. FG: helping in preparing the draft, helping in data analysis. MS: doing statistical analysis, revising the manuscript. MG: designing the study, operating the procedures, data gathering,preparing the draft, revising the manuscript. All the authors have read, revised and confirmed the final version of the manuscript.

## Conflict of Interest

The authors declare that the research was conducted in the absence of any commercial or financial relationships that could be construed as a potential conflict of interest.

## Publisher’s Note

All claims expressed in this article are solely those of the authors and do not necessarily represent those of their affiliated organizations, or those of the publisher, the editors and the reviewers. Any product that may be evaluated in this article, or claim that may be made by its manufacturer, is not guaranteed or endorsed by the publisher.

## References

[1] ESHRE Special Interest Group of Embryology and Alpha Scientists in Reproductive Medicine. The Vienna Consensus: Report of an Expert Meeting on the Development of ART Laboratory Performance Indicators. Reprod BioMed Online (2017) 35(5):494–510. doi: 10.1016/j.rbmo.2017.06.015 28784335

[2] Simón C Martín JC Pellicer A. Paracrine Regulators of Implantation. Baillieres Best Pract Res Clin Obstet Gynaecol (2000) 14(5):815–26. doi: 10.1053/beog.2000.0121 11023802

[3] El-Toukhy T Sunkara S Khalaf Y. Local Endometrial Injury and IVF Outcome: A Systematic Review and Meta-Analysis. Reprod BioMed Online (2012) 25(4):345–54. doi: 10.1016/j.rbmo.2012.06.012 22885017

[4] Frantz S Parinaud J Kret M Rocher-Escriva G Papaxanthos-Roche A Creux H. Decrease in Pregnancy Rate After Endometrial Scratch in Women Undergoing a First or Second *In Vitro* Fertilization. A Multicenter Randomized Controlled Trial. Hum Reprod (2019) 34(1):92–9. doi: 10.1093/humrep/dey334 30496529

[5] Lensen S Osavlyuk D Armstrong S Stadelmann C Hennes A Napier E. A Randomized Trial of Endometrial Scratching Before *In Vitro* Fertilization. N Engl J Med (2019) 380(4):325–34. doi: 10.1056/NEJMoa1808737 30673547

[6] Odendaal J Quenby S. A Randomized Trial of Endometrial Scratching Before *In Vitro* Fertilization. N Engl J Med (2019) 380(18):1777. doi: 10.1056/NEJMc1902642 31042837

[7] Bosteels J Weyers S Puttemans P Panayotidis C Van Herendael B Gomel V. The Effectiveness of Hysteroscopy in Improving Pregnancy Rates in Subfertile Women Without Other Gynaecological Symptoms: A Systematic Review. Hum Reprod Update (2010) 16(1):1–11. doi: 10.1093/humupd/dmp033 19744944

[8] El-Toukhy T Sunkara SK Coomarasamy A Grace J Khalaf Y. Outpatient Hysteroscopy and Subsequent IVF Cycle Outcome: A Systematic Review and Meta-Analysis. Reprod BioMed Online (2008) 16(5):712–9. doi: 10.1016/S1472-6483(10)60486-5 18492377

[9] Pundir J Pundir V Omanwa K Khalaf Y El-Toukhy T. Hysteroscopy Prior to the First IVF Cycle: A Systematic Review and Meta-Analysis. Reprod BioMed Online (2014) 28(2):151–61. doi: 10.1016/j.rbmo.2013.09.025 24365027

[10] Elsetohy KA Askalany AH Hassan M Dawood Z. Routine Office Hysteroscopy Prior to ICSI vs. ICSI Alone in Patients With Normal Transvaginal Ultrasound: A Randomized Controlled Trial. Arch Gynecol Obstet (2015) 291(1):193–9. doi: 10.1007/s00404-014-3397-z 25082070

[11] Takahashi K Mukaida T Tomiyama T Oka C. High Pregnancy Rate After Hysteroscopy With Irrigation in Uterine Cavity Prior to Blastocyst Transfer in Patients Who Have Failed to Conceive After Blastocyst Transfer. Fertil Steril (2000) 74(3):S206. doi: 10.1016/S0015-0282(00)01328-5

[12] Yu HT Weyers S Puttemans P Panayotidis C Van Herendael B Gomel V. The Role of Diagnostic Hysteroscopy Before the First *In Vitro* Fertilization/Intracytoplasmic Sperm Injection Cycle. Arch Gynecol Obstet (2012) 286(5):1323–8. doi: 10.1007/s00404-012-2462-8 22791384

[13] Giorgetti C Terriou P Auquier P Hans E Spach JL Salzmann J. Embryo Score to Predict Implantation After *in-Vitro* Fertilization: Based on 957 Single Embryo Transfers. Hum Reprod (1995) 10(9):2427–31.doi: 10.1093/oxfordjournals.humrep.a136312 8530679

[14] De los Santos MJ Apter S Coticchio G Debrock S Lundin K Plancha CE. Revised Guidelines for Good Practice in IVF Laboratories, (2015). Hum Reprod (2016) 31(4):685–6. doi: 10.1093/humrep/dew016 26908842

[15] Rama Raju GA Shashi Kumari G Krishna KM Prakash GJ Madan K. Assessment of Uterine Cavity by Hysteroscopy in Assisted Reproduction Programme and its Influence on Pregnancy Outcome. Arch Gynecol Obstet (2006) 274(3):160–4. doi: 10.1007/s00404-006-0174-7 16715289

[16] van Hoogenhuijze NE Kasius JC Broekmans FJM Bosteels J Torrance HL. Endometrial Scratching Prior to IVF; Does it Help and for Whom? A Systematic Review and Meta-Analysis. Hum Reprod Open (2019) 2019(1):hoy025. doi: 10.1093/hropen/hoy025 30895265 PMC6396643

[17] van Hoogenhuijze NE Mol F Laven JSE Groenewoud ER Traas MAF Janssen CAH. Endometrial Scratching in Women With One Failed IVF/ICSI Cycle-Outcomes of a Randomised Controlled Trial (SCRaTCH). Hum Reprod (2021) 36(1):87–98. doi: 10.1093/humrep/deaa268 33289528 PMC7801792

[18] Karimzade MA Oskouian H Ahmadi S Oskouian L. Local Injury to the Endometrium on the Day of Oocyte Retrieval has a Negative Impact on Implantation in Assisted Reproductive Cycles: A Randomized Controlled Trial. Arch Gynecol Obstet (2010) 281(3):499–503. doi: 10.1007/s00404-009-1166-1 19568761

[19] Maheux-Lacroix S Dodin S Moore L Bujold E Lefebvre J Bergeron MÈ. Preovulatory Uterine Flushing With Saline as a Treatment for Unexplained Infertility: A Randomised Controlled Trial Protocol. BMJ Open (2016) 6(1):e009897. doi: 10.1136/bmjopen-2015-009897 PMC4716241 26739737

[20] Di Spiezio Sardo A Di Carlo C Minozzi S Spinelli M Pistotti V Alviggi C. Efficacy of Hysteroscopy in Improving Reproductive Outcomes of Infertile Couples: A Systematic Review and Meta-Analysis. Hum Reprod Update (2016) 22(4):479–96. doi: 10.1093/humupd/dmw008 27008893

[21] Potdar N Gelbaya T Nardo LG. Endometrial Injury to Overcome Recurrent Embryo Implantation Failure: A Systematic Review and Meta-Analysis. Reprod BioMed Online (2012) 25(6):561–71. doi: 10.1016/j.rbmo.2012.08.005 23063812

[22] Inagaki N Stern C McBain J Lopata A Kornman L Wilkinson D. Analysis of Intra-Uterine Cytokine Concentration and Matrix-Metalloproteinase Activity in Women With Recurrent Failed Embryo Transfer. Hum Reprod (2003) 18(3):608–15. doi: 10.1093/humrep/deg139 12615834

[23] Johnson NP Bhattu S Wagner A Blake DA Chamley LW. Lipiodol Alters Murine Uterine Dendritic Cell Populations: A Potential Mechanism for the Fertility-Enhancing Effect of Lipiodol. Fertil Steril (2005) 83(6):1814–21. doi: 10.1016/j.fertnstert.2004.11.065 15950655

[24] Johnson NP Farquhar CM Hadden WE Suckling J Yu Y Sadler L. The FLUSH Trial–Flushing With Lipiodol for Unexplained (and Endometriosis-Related) Subfertility by Hysterosalpingography: A Randomized Trial. Hum Reprod (2004) 19(9):2043–51. doi: 10.1093/humrep/deh418 15271870

[25] Lier MCI Özcan H Schreurs AMF van de Ven PM Dreyer K van der Houwen LEE. Uterine Bathing With Sonography Gel Prior to IVF/ICSI-Treatment in Patients With Endometriosis, a Multicentre Randomised Controlled Trial. Hum Reprod Open (2020) 2020(4):hoaa054. doi: 10.1093/hropen/hoaa054 33225080 PMC7668398

[26] Reilly SJ Glanville EJ Dhorepatil B Prentice LR Mol BW Johnson NP. The IVF-LUBE Trial - a Randomized Trial to Assess Lipiodol(®) Uterine Bathing Effect in Women With Endometriosis or Repeat Implantation Failure Undergoing IVF. Reprod BioMed Online (2019) 38(3):380–6. doi: 10.1016/j.rbmo.2018.11.015 30679138

[27] Salehpour S Zamaniyan M Saharkhiz N Zadeh Modares S Hosieni S Seif S. Does Intrauterine Saline Infusion by Intrauterine Insemination (IUI) Catheter as Endometrial Injury During IVF Cycles Improve Pregnancy Outcomes Among Patients With Recurrent Implantation Failure?: An RCT. Int J Reprod BioMed (2016) 14(9):583–8. doi: 10.29252/ijrm.14.9.583 PMC5054295 27738660

